# The socioeconomic distribution of alcohol-related violence in England and Wales

**DOI:** 10.1371/journal.pone.0243206

**Published:** 2021-02-18

**Authors:** Lucy Bryant, Carly Lightowlers

**Affiliations:** 1 The Institute of Alcohol Studies, London, United Kingdom; 2 University of Liverpool, Liverpool, United Kingdom; University of the Witwatersrand, SOUTH AFRICA

## Abstract

Inequalities in alcohol-related health harms have been repeatedly identified. However, the socioeconomic distribution of alcohol-related violence (violence committed by a person under the influence of alcohol)–and of subtypes such as alcohol-related domestic violence–remains under-examined. To examine this, data are drawn from nationally representative victimisation survey, the Crime Survey for England and Wales, from years 2013/14 to 2017/18. Socioeconomic status specific incidence and prevalence rates for alcohol-related violence (including subtypes domestic, stranger, and acquaintance violence) were created. Binomial logistic regressions were performed to test whether the likelihood of experiencing these incidents was affected by socioeconomic status when controlling for a range of pre-established risk factors associated with violence victimisation. Findings generally show lower socioeconomic groups experience higher prevalence rates of alcohol-related violence overall, and higher incidence and prevalence rates for alcohol-related domestic and acquaintance violence. Binomial logistic regression results show that the likelihood of experiencing these types of violence is affected by a person’s socioeconomic status–even when other risk factors known to be associated with violence are held constant. Along with action to address environmental and economic drivers of socioeconomic inequality, provision of publicly funded domestic violence services should be improved, and alcohol pricing and availability interventions should be investigated for their potential to disproportionately benefit lower socioeconomic groups.

## Introduction

*“Inequalities are a matter of life and death*, *of health and sickness*, *of well-being and misery”* [1 p. 16]

The association between alcohol consumption and violence perpetration is long recognised [2–4], with some meta-analyses and longitudinal studies suggesting this may be a causal relationship [2, 5]. National statistics from the Crime Survey for England and Wales (CSEW) in 2017/18 support these findings, with perpetrators in almost two of every five (39%) violent crimes reported by victims as being under the influence of alcohol [6]. However, despite identification of socioeconomic inequalities in alcohol-related health harms [7–9], the socioeconomic distribution of alcohol-related violence (defined as ‘violence committed by a person under the influence of alcohol’ throughout unless otherwise noted) remains under-examined.

### Is low socioeconomic status a risk factor for alcohol-related violence victimisation?

There are three main reasons we might suspect lower socioeconomic status (SES) to be a risk factor for alcohol-related violence. Firstly, whilst alcohol’s various cognitive effects may make violence perpetration more likely [3], sociological work has repeatedly shown that individuals’ responses to intoxicants– including alcohol– can be affected by their surroundings and social context [10–12]. Settings for drinking occasions will likely differ between socioeconomic groups (for example, off-license premises are more densely clustered in lower SES areas [13]) and it is possible we will see different levels of violence experienced by these groups because of this. Secondly, people in lower socioeconomic groups are more likely to be victims of violence overall [14, 15]. This was demonstrated recently in analysis of data from the British Crime Survey (now CSEW) between 2002/03 and 2007/08 showing lower household income to increase risk of violent victimisation [16]. The uneven distribution of violence overall suggests we might see similar inequality in alcohol-related violence victimisation. Finally, a few studies have found a relationship between socioeconomic status and alcohol-related violence, though they disagree whether advantaged or disadvantaged people are at greater risk. Home Office research using the nationally representative British Crime Survey (now CSEW) examined incidents of alcohol-related assaults in the years 1996, 1998 and 2000 and found that rates of alcohol-related stranger assault (“in which the victim did not know any of the offenders” [17 p. 4]) and alcohol-related acquaintance assault (“in which the victim knew one or more of the offenders at least by sight (excluding partners, ex-partners, household members and other relatives)” 17p. 4]) were higher for unemployed adults; those on low household incomes were also found to experience the highest rate of alcohol-related acquaintance assault [17]. Similarly, Scottish hospital data from the early 2000s has shown that alcohol-related facial injuries "disproportionately [affect] young men from socioeconomically deprived areas” [18 p. 644]. Further, in an analysis of alcohol’s harms to others in Wales, a significant association between regional deprivation of a respondent and experience of violent harm as a result of another’s drinking was identified [19]. However, analysis of Australian police data showed higher SES neighbourhoods experienced higher rates of alcohol-related crime, including violence, sexual assault, criminal damage, and anti-social behaviour [20]. Further, the British Crime Survey analysis already discussed found those on higher household incomes experienced the highest rate of alcohol-related stranger assault; private renters were also found to experience higher rates of alcohol-related stranger and acquaintance assaults when compared to social renters [17]. The Australian study seems less reliable than the others because it uses police-recorded crime statistics (which the criminology literature regards as less reliable than victimisation survey and hospital admissions data [21]) and its sub-national sample of only rural communities may not be generalisable.

Mixed findings to date mean we cannot yet confidently conclude that lower socioeconomic status is a risk factor for alcohol-related violence. Further, while some work already discussed has presented findings regarding alcohol-related stranger and acquaintance violence [17], work has yet to disaggregate alcohol-related domestic violence from other subtypes. Population level studies have repeatedly linked alcohol consumption levels to the rates of many subtypes of violence [22], including domestic violence [23], and strong associations have been identified between alcohol consumption by perpetrators and specific types of violence including domestic [23, 24] and stranger violence [17, 25, 26]. Indeed, an evidence summary of meta-analysis and case-control studies of domestic violence and alcohol use concluded alcohol to be a “contributing cause of violence…[contributing] to violence in some people under some circumstances” [23 pp. 423–424]. Failure to disaggregate all of these violence subtypes is not only a substantial limitation, as under-counting of domestic violence incidents has been shown to have distorted official crime trends in recent years [27], but there are reasons why we might suspect the patterns of victimisation across SES groups to vary between violence subtypes. While some have attempted to create all-encompassing theories of violence (e.g. [28]), subtypes of violence are generally recognised to occur in distinct contexts and to have some unique drivers (of which alcohol is just one potential contributory factor). For example, stranger violence is more likely than domestic violence to occur in night-time economy settings in which large volumes of intoxicated individuals cluster together [17, 29]. As already touched upon, evidence suggests a varied propensity of those from different socioeconomic groups to drink (differently) in different contexts (e.g. given that off-trade premises are more densely clustered in lower SES neighbourhoods [13] or that ‘pre-drinking’ patterns in home settings are affected by a person’s SES and motives [30]). Thus, a person’s SES could have diverse relationships with these different forms of violence. This is supported by research examining a nationally representative survey on offending behaviour in England and Wales which found "favoring drinking heavily in pub settings” to be associated with both alcohol-related violence perpetration and lower SES [31 p. 1727].

Finally, a wide range of other risk factors for violence and alcohol-related violence have been identified in previous research, such as sex [14], age [14, 29], attendance of night-time economy spaces [32], and disability [33]. Some such risk factors for violent victimisation may themselves be associated with socioeconomic status. For example, people who live in urban areas are more likely to be victims of violence than those in rural areas [34]–urban areas also have a higher percentage of households living on low incomes [35]. If we hope to design policy action to address socioeconomic inequalities in alcohol-related violence, we need to understand if SES impacts upon the probability of alcohol-related violence once such factors have been accounted for.

## Materials and methods

### Design

This study combines five waves of data drawn from the Crime Survey for England and Wales for years 2013/14 to 2017/18 [36–40] employing a cross-sectional between-subjects design to:

- create and compare prevalence rates (the percentage of people who experienced a given crime in a year) and incidence rates (the number of incidents of a given crime in a year per 1000 people) of alcohol-related violence overall, as well as alcohol-related domestic, stranger and acquaintance violence, for different socioeconomic groups, and;
- perform binomial logistic regression analyses to confirm the effect of a range of other risk factors associated with violence on any relationship identified.

This survey is nationally representative of the household population in England and Wales, and is administered face-to-face to more than 35,000 adults, identified from a random sample of addresses, annually (further detailed description of the sampling strategy employed can be found at [41]). Respondents are asked about their victimisation within the last 12 months, as well as information on their employment, income, and housing. Response rates to this survey have remained between 70–75% since the 2008/09 release [42].

### Procedure

Each year, information from respondents to the Crime Survey for England and Wales is held across two datasets: the Victim Form and the Non-Victim Form datasets. Each row of the Non-Victim Form dataset contains information on an individual respondent, such as measures of their socioeconomic status. Each row of the Victim Form dataset contains detail on an individual instance of crime or a series of instances of the same crime, including, if the incident was violence and what type of violence it was– domestic, stranger, or acquaintance (Table 1 shows which variables relating to this work are contained in each dataset). In order to analyse details of a crime and its victim together, these datasets are merged. This is achieved by appending respondent characteristics (from the Non-Victim Form dataset) to the incident or crime series data (in the Victim Form dataset) via a unique ’Case identifier’ contained in each. Using this method, all records were matched accurately without duplication.

**Table 1:** Measures used from each CSEW dataset.

	Non-Victim Form dataset	Victim Form dataset
**SES**	Total household income	None
	Housing tenure of respondents	
	Occupation of respondents	
**Alcohol-related violence subtypes (e.g. domestic)**	None	Measure indicating what type of violence an incident was (domestic, stranger, or acquaintance) and another showing whether the offender was under the influence of alcohol
**Respondent risk factors**	Sex	None
	Age	
	Rural or urban home location	
	Whether respondent has a disability	
	Frequency respondent visits clubs and pubs	

Further, given the relatively rare nature of violent events, a large sample was required in order to assure sufficient cases of violence for analysis. To this end, data were pooled from five years in order to increase the reliability and accuracy of any results. The final sample thus totalled at 174,178, including 1398 incidents (unweighted) of alcohol-related violence. Weighting variables used ensure sample is nationally representative, by (amongst other things) "[compensating] for unequal address selection probabilities" as well as "[adjusting] for differential non-response” [41 p. 97], and to create estimates of how many victims and incidents of each kind there were across the whole population. Further details of the full weighting procedure are available in the CSEW User Guide [41]. Previously, such weighting included a cap of five on the incidents of one kind that could be reported by a respondent, as a method to remove outliers. This led to undercounting of violence–particularly domestic violence–and a method developed by Walby, Towers, and Francis [27] was needed to remove this capping and more accurately count these incidents. However, this undercounting has since been addressed through changes to the weighting variables, using “the 98th percentile of victim incident counts for each crime type (calculated over several years)” to cap repeat victimisation reports, avoiding the undercounting problem potentially encountered by previous work of this kind [41 p. 14].

### Measures

#### Violence

Respondents of the CSEW are asked about their experiences of a range of crimes–including violence–in the 12 months prior to the interview. Incidents of violence are described to interviewers through a series of questionnaire items. Wounding (“the incident results in severe or less serious injury, for example, cuts, severe bruising, chipped teeth, bruising or scratches requiring medical attention or any more serious injuries” [41 p. 44]), assault with minor injury (“an incident where the victim was punched, kicked, pushed or jostled and the incident resulted in minor injury to the victim, for example, scratches or bruises” [41 p. 44]) and violence without injury (“an incident (or attempt) where the victim was punched, kicked, pushed or jostled but resulted in no injury” [41 p. 44]) are coded by trained crime survey coders [41] as domestic violence (incidents “that involve partners, ex-partners, other relatives or household members” [41 p. 44]), stranger violence (incidents “in which the victim did not have any information about the offender(s), or did not know and had never seen the offender(s) before” [41 p. 44]), or acquaintance violence (incidents “in which the victim knew one or more of the offenders, at least by sight; it does not include domestic violence” [41 p. 44]). Respondents are also asked whether they believed their perpetrators were under the influence of alcohol (the full questionnaire is published with the survey data annually [36–40]). The variable ‘Whether offender was under the influence of drink’ indicates if an incident was alcohol-related (Table 2), and the variable ‘CSEW Type of violence’ indicates whether an incident or series of crimes was violent, and whether it was classed as domestic, stranger or acquaintance violence (Table 3). While it is recognised that domestic violence can comprise other elements beyond physical harm (e.g. verbal and psychological harms), the measure used in this work is limited to physical violence.

**Table 2:** Whether offender was under the influence of drink (weighted).

	*Frequency*	*Percent*
*Yes*	9455300	30.3
*No*	15258354	49.0
*Don’t know*	6444351	20.7
*Total*	31158005	100.0

Base (n = 15315, unweighted) = sub-sample of victim form sample, item presented to participants for the first three incidents or series of incidents they describe only, and excluding incidents where the victim was unable to comment on the perpetrator, or the perpetrator was 10 years of age or younger (n = 35722, unweighted, 70.0% of victim form sample). In analysis those responding ‘Don’t know’ were also marked as missing.

**Table 3:** CSEW type of violence (weighted).

	*Frequency*	*Percent*
*Domestic*	2148897	20.7
*Stranger*	3637083	35.1
*Acquaintance*	4577872	44.2
*Total*	10363853	100.0

Base (n = 3396, unweighted) = whole victim form sample, excluding those marked missing (non-violent incidents, and incidents not defined as domestic, stranger, or acquaintance violence (for example, robbery), n = 47641, unweighted, 93.3% of victim form sample).

#### Socioeconomic status

Three household and individual level variables are used here with which to explore SES: total household income; housing tenure; and occupation of respondent. These individual and household measures have previously been successfully deployed in analysis of violence and SES [43, 44]. The limitations of using such indicators in isolation has been demonstrated [45], and so analysis is repeated here with this selection of SES measures in order to triangulate the findings.

Violence risk factors: a range of risk factors associated with violence (as demonstrated in the introduction) are included as control variables (in binary form) in the second part of this analysis. These are outlined in Table 1. Information on the derivation of and frequency tables for these variables are included in the (S1 to S6 Tables in S1 File):

- Respondent sex
- Respondent age (converted to a binary variable to maintain statistical power, those over 30 years and those 30 or under)
- Whether respondent lives in a rural or urban area
- Whether respondent has a disability
- Frequency respondent visits clubs (in the last month or not) and pubs (weekly and upwards, or less)

### Analysis

#### a) Creating incidence rates

Total incident figures for each type of alcohol-related violence were derived from two variables; ‘CSEW Type of violence’ and ‘whether offender was under the influence of drink’. These were calculated for each socioeconomic group within each SES variable, using a weighted dataset comprising all victim form datasets, with SES information on respondents appended from the non-victim form datasets, for the period analysed. From this, population figures for the various socioeconomic groups as presented in Tables 4–6 were used to create incidence rates. In each wave of the survey, respondents reported their victimisation (if any) for the previous 12 months. Therefore, the incidence rate throughout this work is the average annual incidence rate for the period 2013/14 to 2017/18.

**Table 4:** Total household income (weighted).

	*Frequency*	*Percent*
*£19,999 and under*	67463743	33.8
*£20,000 to £39,999*	61471016	30.8
*£40,000 and up*	70596463	35.4
*Total*	199531222	100.0

Base (n = 151562, unweighted) = whole sample, excluding uncategorisable responses marked as missing (n = 22616, unweighted, 13.0% of whole sample). Response categories condensed from 7 categories (Under £10,000, £10,000-£14,999, £15,000-£19,999, £20,000-£29,999, £30,000-£39,999, £40,000 to £49,999 and £50,000 or more) to improve statistical power.

**Table 5:** Housing tenure of respondents (weighted).

	*Frequency*	*Percent*
*Social renters*	34119598	15.0
*Private renters*	50372991	22.1
*Owners*	143417329	62.9
*Total*	227909919	100.0

Base (n = 173249, unweighted) = whole sample, excluding uncategorisable responses marked as missing (n = 929, unweighted, 0.5% of whole sample).

**Table 6:** Occupation of respondents (weighted).

	*Frequency*	*Percent*
*Never worked or long term unemployed*	8853988	4.1
*Routine and manual*	76098362	35.6
*Intermediate*	51228390	24.0
*Managerial and professional*	77625867	36.3
*Total*	213806608	100.0

Base (n = 167434, unweighted) = whole sample, excluding uncategorisable responses marked missing (n = 6744, unweighted, 3.9% of whole sample). Details of the occupations included in these categories can be found here: https://www.ons.gov.uk/methodology/classificationsandstandards/otherclassifications/thenationalstatisticssocioeconomicclassificationnssecrebasedonsoc2010.

#### b) Creating prevalence rates

By cross-referencing the ‘CSEW Type of violence’ and ‘whether offender was under the influence of drink’ variables, all incidents contained in all victim form datasets were marked as whether they were alcohol-related violence, and whether they were alcohol-related domestic, stranger, or acquaintance violence. These datasets were merged with all the non-victim form datasets for the period covered. Each respondent was now marked as a non-victim or victim of alcohol-related violence overall, as well as of each subtype of alcohol-related violence, and total weighted victim counts were created. From this, population figures for the various socioeconomic groups as presented in Tables 4–6 were used to create prevalence rates. In each wave of the survey, respondents reported their victimisation (if any) for the previous 12 months. Therefore, the prevalence rate referred to throughout this work is the average annual prevalence rate for the period 2013/14 to 2017/18. Two-tailed chi-squared tests were also performed to examine the association between socioeconomic status and alcohol-related violence victimisation.

#### c) Regression analyses

Binomial logistic regression analyses were performed on weighted data from the combined non-victim form dataset created in step (b). Twelve binary logistic regression analyses were performed sequentially in total: using one of the three measures of socioeconomic status as an independent variable, against each of the four binary violence outcome variables (alcohol-related violence, alcohol-related domestic violence, alcohol-related stranger violence and alcohol-related acquaintance violence) as a dependent variable. In all 12 models the previously outlined risk factors for violent victimisation (age, sex, night-time economy attendance, whether the respondent has a disability, living in an urban or rural setting) were controlled for. Given the possibility of incorrectly rejecting a true null hypothesis (Type I error), with the number of simultaneously tested hypotheses here, the Bonferroni correction was applied, adjusting our significance threshold from an original value of p<0.05 to p<0.004.

All analysis was performed using SPSS v 24. As this work comprises secondary analysis of government published data, ethical approval was not sought for this analysis; data are received pre-anonymised, with consent of participants having already been obtained. Further, the data owner (Office for National Statistics) has pre-approved the reporting of these findings to void any possibility of disclosure.

## Results

### 1. Alcohol-related violence overall

Across the whole sample, the prevalence rate for alcohol-related violence overall was 0.87%, while the incidence rate was 19.1 incidents per 1000 of the population. Lower socioeconomic groups experienced higher prevalence rates of alcohol-related violence overall (total household income: χ2 = 35922.96, p<0.001; housing tenure: χ2 = 523448.76, p<0.001; occupation: χ2 = 47003.53, p<0.001). For two of the three socioeconomic status indicators used, the lowest groups experienced the highest prevalence rates for alcohol-related violence overall; 1.07% for those in households earning £19,999 and under and 1.01% for the group ‘Never worked and long term unemployed’ (see Table 7).

**Table 7:** Alcohol-related violence experienced in last 12 months by socioeconomic status, 2013/14-2017/18 (weighted).

	Alcohol-related violence		Alcohol-related domestic violence		Alcohol-related stranger violence		Alcohol-related acquaintance violence	
	Prevalence rate*	Incidence rate	Prevalence rate*	Incidence rate	Prevalence rate*	Incidence rate	Prevalence rate*	Incidence rate
	% (95% CI)	per 1000 (95% CI)	% (95% CI)	per 1000 (95% CI)	% (95% CI)	per 1000 (95% CI)	% (95% CI)	per 1000 (95% CI)
Total household income								
£19,999 and under	1.07%	20.982	0.19%	5.362	0.49%	6.311	0.40%	9.309
	(1.065%-1.071%)	(20.940–21.023)	(0.193%-0.195%)	(5.342–5.383)	(0.485%-0.489%)	(6.288–6.334)	(0.402%-0.406%)	(9.281–9.336)
£20,000 to £39,999	0.84%	15.49	0.09%	1.399	0.47%	8.007	0.30%	6.083
	(0.833%-0.839%)	(15.452–15.527)	(0.091%-0.093%)	(1.388–1.411)	(0.469%-0.473%)	(7.980–8.034)	(0.294%-0.297%)	(6.060–6.107)
£40,000 and above	0.78%	24.268	0.06%	2.718	0.53%	14.001	0.20%	7.549
	(0.777%-0.782%)	(24.225–24.312)	(0.061%-0.063%)	(2.703–2.732)	(0.531%-0.535%)	(13.968–14.034)	(0.198%-0.201%)	(7.525–7.574)
	*χ2 = 35922.96, df = 2, p<0.001**, Cramer’s V = 0.013		*χ2 = 56130.26, df = 2, p<0.001**, Cramer’s V = 0.017		*χ2 = 2838.77, df = 2, p<0.001**, Cramer’s V = 0.004		*χ2 = 48341.69, df = 2, p<0.001**, Cramer’s V = 0.016	
Housing tenure								
Social renters	1.28%	29.603	0.26%	12.132	0.52%	6.75	0.52%	10.721
	(1.272%-1.281%)	(29.534–29.672)	(0.259%-0.263%)	(12.087–12.176)	(0.515%-0.521%)	(6.717–6.784)	(0.513%-0.519%)	(10.679–10.763)
Private renters	1.53%	33.734	0.17%	2.573	0.92%	15.415	0.48%	15.745
	(1.525%-1.533%)	(33.673–33.794)	(0.172%-0.175%)	(2.557–2.590)	(0.912%-0.918%)	(15.374–15.456)	(0.476%-0.480%)	(15.704–15.787)
Owners	0.53%	11.473	0.05%	0.85	0.33%	7.529	0.15%	3.094
	(0.523%-0.526%)	(11.452–11.494)	(0.052%-0.053%)	(0.844–0.855)	(0.330%-0.333%)	(7.512–7.546)	(0.149%-0.151%)	(3.083–3.105)
	*χ2 = 523448.76, df = 2, p<0.001**, Cramer’s V = 0.048		*χ2 = 131153.69, df = 2, p<0.001**, Cramer’s V = 0.024		*χ2 = 262084.90, df = 2, p<0.001**, Cramer’s V = 0.034		*χ2 = 227680.95, df = 2, p<0.001**, Cramer’s V = 0.032	
Occupation								
Never worked or long term unemployed	1.01%	23.87	0.21%	2.686	0.40%	5.167	0.40%	16.017
	(1.000%-1.015%)	(23.748–23.992)	(0.205%-0.212%)	(2.645–2.727)	(0.393%-0.403%)	(5.110–5.224)	(0.396%-0.406%)	(15.917–16.117)
Routine or manual	0.93%	19.47	0.16%	5.931	0.48%	7.67	0.32%	5.87
	(0.929%-0.934%)	(19.433–19.508)	(0.157%-0.159%)	(5.910–5.951)	(0.474%-0.478%)	(7.646–7.693)	(0.322%-0.325%)	(5.849–5.890)
Intermediate	0.83%	26.913	0.09%	1.627	0.48%	11.287	0.28%	13.998
	(0.826%-0.831%)	(26.859–26.967)	(0.089%-0.091%)	(1.614–1.641)	(0.474%-0.478%)	(11.252–11.322)	(0.274%-0.277%)	(13.959–14.037)
Managerial or professional	0.64%	12.731	0.08%	1.241	0.42%	9.327	0.15%	2.163
	(0.637%-0.641%)	(12.701–12.761)	(0.078%-0.080%)	(1.232–1.251)	(0.418%-0.421%)	(9.301–9.352)	(0.151%-0.153%)	(2.151–2.175)
	*χ2 = 47003.53, df = 3, p<0.001**, Cramer’s V = 0.015		*χ2 = 30698.37, df = 3, p<0.001**, Cramer’s V = 0.012		*χ2 = 4026.12, df = 3, p<0.001**, Cramer’s V = 0.004		*χ2 = 55329.04, df = 3, p<0.001**, Cramer’s V = 0.016	

Base (n = income: 151562; tenure: 173249; occupation: 167434, unweighted): whole sample, excluding respondents marked missing for respective socioeconomic status variables (see Tables 4–6). Confidence intervals are calculated using a design factor for 1.2.

**Significant based on threshold of p<0.05.

#### 1a. Alcohol-related domestic and acquaintance violence

Similar patterns are seen when disaggregating patterns in alcohol-related domestic and alcohol-related acquaintance violence. For all socioeconomic measures, prevalence rates for alcohol-related domestic violence were highest for the lowest socioeconomic group (total household income: χ2 = 56130.26, p<0.001; housing tenure: χ2 = 131153.69, p<0.001; occupation: χ2 = 30698.37, p<0.001). For housing tenure, the prevalence rate for the lowest socioeconomic group (social renters, 0.26%) was more than five times that of the highest (owners, 0.05%). Incidence rates were highest for the lowest socioeconomic group in two measures, with the most dramatic disparity seen between the incidence rates of alcohol-related domestic violence when measuring socioeconomic status through housing tenure; the lowest group (social renters) had an incidence rate more than 14 times as high as the highest group (owners). Similarly, the lowest socioeconomic groups experienced the highest prevalence (total household income: χ2 = 48341.69, p<0.001; housing tenure: χ2 = 227680.95, p<0.001; occupation: χ2 = 55329.04, p<0.001) and incidence rates for alcohol-related acquaintance violence, except social renters whose incidence rate was not as high as that of private renters (those in households earning £19,999 and under, 0.40% prevalence and 9.31 incidents per 1000 people; social renters, 0.52% prevalence and 10.72 incidents per 1000 people; those unemployed, 0.40% prevalence and 16.02 incidents per 1000 people).

#### 1b. Alcohol-related stranger violence

There are no clear trends in the prevalence and incidence rates for alcohol-related stranger violence across socioeconomic measures (see Table 7). Prevalence and incidence rates were highest for those earning £40,000 and above (0.53%, and 14.00 incidents per 1000 people), private renters (0.92%, and 15.41 incidents per 1000 people), and those with occupations classed as intermediate (0.48% (joint with routine and manual workers) and 11.29 incidents per 1000 people).

### 2. Influence of other risk factors

Binomial logistic regression results show that those in lower socioeconomic groups are more likely than others to experience alcohol-related violence overall when other known risk factors for violence are held constant. In some cases, including additional known risk factors brought this relationship into sharper relief: whereas social renters were found to experience lower prevalence and incidence rates of alcohol-related violence than private renters, when additional violence risk factors were included in the analysis, social renters were more than twice as likely as owners to experience this [OR = 2.117, 95% CI = (2.109–2.126), reference category: housing tenure, owners], while private renters were only around one and a half times as likely as owners to [OR = 1.448, 95% CI = (1.443–1.453), reference category: housing tenure, owners]. For all socioeconomic variables used, the lowest socioeconomic group were more likely than the highest group to experience this violence (e.g. those ‘never worked or long term unemployed’ [OR = 1.456, 95% CI = (1.445–1.467)], reference category: managerial or professional occupation), as were all central socioeconomic groups (e.g. those in intermediate occupations [OR = 1.403, 95% CI = (1.397–1.409)], reference category: managerial or professional occupation). Being male, 30 or under, visiting the pub weekly or more, having visited a nightclub in the last month, or having a disability were all found to also increase a person’s risk of experiencing alcohol-related violence, in all the analyses performed (see Tables 8–10). As the weighting variables provided in the dataset serve to create population level estimates, results of the same regressions performed on unweighted data as a sensitivity analysis are presented in the S7–S9 Tables in S1 File, to verify that statistical significance was not an artefact of this.

**Table 8:** Binomial logistic regression of association between socioeconomic status (measured by total household income), demographic and violence risk factor variables, and experience of alcohol-related violence and subtypes, run on weighted data.

	Experienced alcohol-related violence?	Experienced alcohol-related domestic violence?	Experienced alcohol-related stranger violence?	Experienced alcohol-related acquaintance violence?
	Odds ratio (95% CI)	Odds ratio (95% CI)	Odds ratio (95% CI)	Odds ratio (95% CI)
Female	0.498*	1.765*	0.287*	0.636*
	(0.496–0.500)	(1.750–1.781)	(0.286–0.289)	(0.633–0.639)
Urban	1.039*	0.958*	1.158*	0.861*
	(1.034–1.043)	(0.948–0.969)	(1.151–1.165)	(0.855–0.867)
Age (over 30)	0.315*	0.475*	0.300*	0.288*
	(0.314–0.316)	(0.471–0.480)	(0.298–0.301)	(0.286–0.289)
Visited pub weekly or more in last month	1.502*	1.421*	1.609*	1.369*
	(1.497–1.507)	(1.406–1.436)	(1.601–1.616)	(1.361–1.377)
Visited nightclub/disco in last month	2.790*	1.772*	3.281*	2.433*
	(2.780–2.801)	(1.751–1.794)	(3.265–3.297)	(2.417–2.449)
Respondent has a disability	1.935*	3.138*	1.228*	2.740*
	(1.928–1.942)	(3.110–3.165)	(1.221–1.235)	(2.725–2.756)
Total household income: £19,999 and under**	1.270*	2.403*	0.911*	1.729*
	(1.265–1.274)	(2.376–2.430)	(0.907–0.916)	(1.717–1.740)
Total household income: £20,000 to £39,999**	1.133*	1.352*	0.981*	1.510*
	(1.128–1.137)	(1.335–1.369)	(0.976–0.986)	(1.499–1.520)

Experienced alcohol-related violence: Yes, n = 1179, unweighted; Experienced alcohol-related domestic violence: Yes, n = 213, unweighted; Experienced alcohol-related stranger violence: Yes, n = 580, unweighted; Experienced alcohol-related acquaintance violence: Yes, n = 410, unweighted. Base for all, n = 150570, unweighted = whole sample excluding respondents marked missing for total household income variable or other demographic and violence risk factor variables (n = 23608, 13.6% of whole sample).

*Significant based on threshold adjusted through Bonferroni correction to p<0.004, from an original value of p<0.05.

**Reference category: Total household income: £40,000 and above.

**Table 9:** Binomial logistic regression of association between socioeconomic status (measured by housing tenure), demographic and violence risk factor variables, and experience of alcohol-related violence and subtypes, run on weighted data.

	Experienced alcohol-related violence?	Experienced alcohol-related domestic violence?	Experienced alcohol-related stranger violence?	Experienced alcohol-related acquaintance violence?
	Odds ratio (95% CI)	Odds ratio (95% CI)	Odds ratio (95% CI)	Odds ratio (95% CI)
Female	0.489*	1.937*	0.281*	0.620*
	(0.487–0.490)	(1.921–1.953)	(0.280–0.283)	(0.617–0.624)
Urban	0.980*	0.956*	1.097*	0.776*
	(0.976–0.984)	(0.946–0.966)	(1.091–1.103)	(0.771–0.781)
Age (over 30)	0.342*	0.488*	0.317*	0.339*
	(0.340–0.343)	(0.483–0.493)	(0.316–0.319)	(0.337–0.341)
Visited pub weekly or more in last month	1.571*	1.576*	1.643*	1.430*
	(1.565–1.576)	(1.561–1.592)	(1.636–1.650)	(1.422–1.438)
Visited nightclub/disco in last month	2.783*	1.809*	3.211*	2.468*
	(2.773–2.794)	(1.789–1.830)	(3.196–3.227)	(2.452–2.484)
Respondent has a disability	1.786*	3.284*	1.108*	2.529*
	(1.780–1.793)	(3.257–3.312)	(1.102–1.114)	(2.515–2.543)
Housing tenure: social renters**	2.117*	3.678*	1.449*	2.816*
	(2.109–2.126)	(3.641–3.715)	(1.440–1.457)	(2.797–2.834)
Housing tenure: private renters**	1.448*	2.295*	1.220*	1.692*
	(1.443–1.453)	(2.271–2.320)	(1.215–1.226)	(1.681–1.702)

Experienced alcohol-related violence: Yes, n = 1286, unweighted; Experienced alcohol-related domestic violence: Yes, n = 230, unweighted; Experienced alcohol-related stranger violence: Yes, n = 639, unweighted; Experienced alcohol-related acquaintance violence: Yes, n = 443, unweighted. Base for all, n = 171775, unweighted = whole sample excluding respondents marked missing for housing tenure variable or other demographic and violence risk factor variables (n = 2403, 1.4% of whole sample).

*Significant based on threshold adjusted through Bonferroni correction to p<0.004, from an original value of p<0.05.

**Reference category: Housing tenure: owners.

**Table 10:** Binomial logistic regression of association between socioeconomic status (measured by occupation), demographic and violence risk factor variables, and experience of alcohol-related violence and subtypes, run on weighted data.

	Experienced alcohol-related violence?	Experienced alcohol-related domestic violence?	Experienced alcohol-related stranger violence?	Experienced alcohol-related acquaintance violence?
	Odds ratio (95% CI)	Odds ratio (95% CI)	Odds ratio (95% CI)	Odds ratio (95% CI)
Female	0.514*	1.755*	0.287*	0.690*
	(0.513–0.516)	(1.740–1.769)	(0.285–0.288)	(0.686–0.694)
Urban	1.064*	1.123*	1.175*	0.841*
	(1.060–1.069)	(1.111–1.136)	(1.168–1.182)	(0.836–0.847)
Age (over 30)	0.285*	0.368*	0.267*	0.290*
	(0.284–0.286)	(0.365–0.372)	(0.266–0.268)	(0.288–0.291)
Visited pub weekly or more in last month	1.566*	1.360*	1.618*	1.562*
	(1.561–1.572)	(1.347–1.374)	(1.611–1.625)	(1.552–1.572)
Visited nightclub/disco in last month	2.544*	2.102*	2.844*	2.273*
	(2.534–2.554)	(2.078–2.127)	(2.830–2.859)	(2.256–2.289)
Respondent has a disability	1.841*	3.286*	1.137*	2.634*
	(1.834–1.847)	(3.258–3.314)	(1.131–1.144)	(2.618–2.649)
Occupation: never worked or long term unemployed**	1.456*	1.642*	1.069*	2.180*
	(1.445–1.467)	(1.613–1.671)	(1.057–1.081)	(2.153–2.207)
Occupation: routine or manual occupation**	1.299*	1.677*	1.013*	1.900*
	(1.294–1.304)	(1.660–1.693)	(1.008–1.018)	(1.887–1.914)
Occupation: intermediate occupation**	1.403*	1.063*	1.326*	1.832*
	(1.397–1.409)	(1.050–1.076)	(1.319–1.334)	(1.817–1.846)

Experienced alcohol-related violence: Yes, n = 1194, unweighted; Experienced alcohol-related domestic violence: Yes, n = 224, unweighted; Experienced alcohol-related stranger violence: Yes, n = 586, unweighted; Experienced alcohol-related acquaintance violence: Yes, n = 408, unweighted. Base for all, n = 166034, unweighted = whole sample excluding respondents marked missing for occupation variable or other demographic and violence risk factor variables (n = 8144, 4.7% of whole sample).

*Significant based on threshold adjusted through Bonferroni correction to p<0.004, from an original value of p<0.05.

**Reference category: Occupation: managerial or professional occupation.

Binomial logistic regression results also show that those in lower socioeconomic groups are more likely than others to experience the subtypes of alcohol-related domestic and acquaintance violence, when other known risk factors for violence are held constant. For all socioeconomic variables, the lowest and central socioeconomic groups were more likely than the highest group to experience these forms of violence. Further, in some cases, the contribution of socioeconomic status to a person’s risk here is sizeable. Considering alcohol-related domestic violence, a social renter is more than three and a half times as likely to experience this than a home owner [OR = 3.678, 95% CI = (3.641–3.715), reference category: housing tenure, owners] while those in households earning £19,999 or less are almost two and a half times as likely to [OR = 2.403, 95% CI = 2.376–2.430), reference category: total household income, £40,000 and above]. It should also be noted that having a disability also raised a person’s risk of experiencing alcohol-related domestic violence by more than three times, in all regression models presented (see Tables 8–10). In two of the three regression models presented (Tables 8 and 10), having a disability raised a person’s risk of experiencing alcohol-related domestic violence to a greater degree than the already sizeable effect of belonging to the lowest SES group.

The regression results for alcohol-related stranger violence diverge from these other violence subtypes (see Tables 8–10). In some cases the lower socioeconomic groups were in fact protected from this violence by their status; lower income groups were less likely to experience this violence than the highest group, those earning £40,000 and over (those in households earning £19,999 or less [OR = 0.911, 95% CI = (0.907–0.916), reference category: total household income, £40,000 and above] and those earning between £20,000 and £39,999 [OR = 0.981, 95% CI = (0.976–0.986), reference category: total household income, £40,000 and above]. In the case of occupation, the lowest group were statistically more likely to experience this violence than the highest group–but by less than 10% [OR = 1.069, 95% CI = (1.057–1.081), reference category: managerial or professional occupation]. Along with these small or protective effect sizes, it should be noted that in the unweighted regressions included in (S7–S9 Tables in S1 File), income and occupation were not found to be significant predictors of alcohol-related stranger violence. All other risk factors–being aged 30 and under, male, living in an urban area, having a disability– were found to significantly increase this risk; particularly night-time economy attendance. A person’s likelihood of experiencing alcohol-related stranger violence was raised by weekly pub and monthly nightclub attendance, sometimes by as much as three times (e.g. see Table 8, those attending nightclubs monthly [OR = 3.281, 95% CI = (3.265–3.297), reference category: no nightclub attendance in last month], as well as those who visited the pub weekly or more [OR = 1.609, 95% CI = (1.601–1.616), reference category: visited pubs less than once a week]). Those same night-time economy visits had a smaller effect on all other kinds of alcohol-related violence, however regular nightclub attendance also more than doubled a person’s likelihood of experiencing alcohol-related acquaintance violence (e.g. see Table 9, [OR = 2.468, 95% CI = (2.452–2.484), reference category: no nightclub attendance in last month]).

## Discussion

Our results suggest that being of a lower socioeconomic status is a risk factor for experiencing alcohol-related violent victimisation, and particularly for alcohol-related domestic and acquaintance violence. The finding of lower socioeconomic status as a risk factor for alcohol-related violence reflects patterns identified for violent victimisation in general [14, 15], and the inequalities in alcohol-related health harms experienced between socioeconomic groups [7–9]. This finding corroborates previous work examining hospital admissions for alcohol-related facial injuries in Scotland, which found those in the most deprived regions were more than six times as likely to be injured in this way when compared to the most advantaged regions [18]. The finding that this disparity in overall alcohol-related violence comprises wide disparities in alcohol-related domestic and acquaintance violence is novel and holds important implications for policy decisions moving forward.

These findings represent a notable development in the understanding of the distribution of alcohol-related violence. It is important to consider this in light of some limitations of this study, not least those associated with the use of victimisation surveys. The Crime Survey for England and Wales remains an internationally recognised source for crime statistics [46] and, unlike police recorded crime statistics, holds the designation of official statistics in England and Wales from the National Statistics Authority [47]. While victimisation survey data are generally accepted in criminology to improve substantially on police-recorded crime data as a measure of crime levels, survey data are not without its own limitations e.g. recall error [21, 42] or difficulty respondents may have in identifying perpetrator intoxication. However, there have been measures introduced to ensure some such limitations are minimised within the Crime Survey for England and Wales. Importantly, detailed reports of incidents from respondents are coded as violent or otherwise by trained coders, minimising categorisation errors respondents might make. Much of the criticism surrounding the Crime Survey for England and Wales has instead focused on its sampling, with suggestions that it has under-sampled lower socioeconomic groups [48]; a limitation that will, if anything, underplay the results we have found in underestimating the strength of the association between lower SES groups and their increased probability of experiencing of alcohol-related violence. Further detail of the survey methodology can be found in the CSEW user guide [41]. Further, whilst the importance of interactions between individual and neighbourhood level socioeconomic status indicators in investigation of alcohol consumption patterns have been demonstrated in other studies [49–51], exploring such interactions was beyond the scope of the paper. We encourage such analysis be taken forward to illuminate contributions of neighbourhood level socioeconomic status.

Notwithstanding these limitations, this study represents the first of its kind to disaggregate subtypes of alcohol-related violence, including alcohol-related domestic violence, in order to understand their distribution across SES groups. We consider these findings to build on links between poverty and domestic violence victimisation that have been identified in other literature [24]– particularly the uneven impact of the global economic crisis in 2008 on levels of violence experienced by different groups. Walby, Towers, and Francis noted that the crisis has “reduced income levels and increased inequalities and thereby reduced the propensity of victims to escape violence” [27 p. 1228]. Returning to our findings of highly disproportionate rates of alcohol-related domestic violence for lower SES groups, it is possible that these effects are amplified as alcohol sales outlets are more heavily clustered in lower SES neighbourhoods [13] and alcohol availability is linked to levels of violence. For example, research from Scotland has shown rates of violence to be "consistently and significantly higher in areas with more alcohol outlets”, for both on- and off-sales [52 p. 8]. While not the focus of this work, it is also important to touch upon the findings relating to disability and alcohol-related domestic violence. In each model, having a disability increased a person’s risk of alcohol-related domestic violence by more than three times–stronger than the already sizeable effect of belonging to the lowest socioeconomic group for two of the three SES measures used (household income and occupation). Previous research has identified disability as a risk factor for domestic violence [53], and this finding relating to alcohol-related domestic violence expands this understanding. These findings are important and warrant further investigation–particularly as the limitations of analysis using aggregate population data, as was the case here, in examining violence against disabled people have been noted [54]. One avenue of future research this study might inform is the confluence of disability and lower SES, and how this affects alcohol-related domestic violence victimisation; as it has been noted that perpetrators of domestic violence may intentionally restrict disabled victims’ financial resources [55 as cited in 54].

The finding of no consistent relationship between alcohol-related stranger violence and SES when other violence risk factors were held constant refines findings from previous work; analysis of British Crime Survey data from the years 1996, 1998 and 2000 found those unemployed to have an incidence rate of alcohol-related stranger violence 2.6 times higher than those in employment [17]. Our findings suggest this incidence rate was confounded by other factors; possibly night-time economy attendance. Our results suggest exposure to night-time economy settings increases the likelihood of experiencing alcohol-related violence; specifically, alcohol-related stranger violence. Regular nightclub attendance (once a month at least) as much as trebled a person’s likelihood of experiencing alcohol-related stranger violence, and weekly pub trips raised a person’s risk more for alcohol-related stranger violence than for any other violence subtype. This corroborates previous research which has identified an association between experiencing violence and night-time economy attendance [56]. Indeed, the same British Crime Survey analysis discussed previously found that the incidence of alcohol-related stranger violence amongst those attending nightclubs between one to three times in the last month was more than twice as high as in the general population, and amongst those visiting a pub nine times or more in the last month (roughly twice a week), more than three and a half times as high [17]. Considering that the socioeconomic disparities found in overall alcohol-related violence comprise wide disparities in alcohol-related domestic and acquaintance violence, but that night-time economy attendance is a greater risk factor for alcohol-related stranger violence, this suggests policy interventions focused on night-time economy settings, such as business best practice schemes (e.g. PubWatch [57] or Best Bar None [58] in the UK), will have little impact on the inequalities presented in this paper.

This discussion highlights important considerations for policy and future research concerned with violence prevention and alcohol harm, as well as socioeconomic equality more generally. First, in light of findings in this paper, we implore an immediate improvement in the provision of and access to publicly funded domestic violence services within lower SES neighbourhoods. There has been a chronic under-provision of both domestic violence and alcohol treatment services in this country for many years [59, 60], and this should be addressed nationwide for many reasons. We suggest the findings of this paper prompt a focused increase in provision and access to (e.g. consideration of transport or childcare needs etc.) domestic violence services for lower SES neighbourhoods specifically, because this research indicates how this alcohol-related harm is distributed. There are likely many factors contributing to this inequality (for example, see the extensive literature examining the alcohol health harm paradox [7]), and so we should address the pattern of harm directly as a matter of urgency. As recommended by the National Institute for Health and Care Excellence [61], it would also be beneficial for these services to continue to build their awareness and understanding of alcohol-related domestic violence victimisation and to improve links to other service providers through multi-agency working.

We further urge policymakers to consider SES as an important contextual factor in shaping the relationship between alcohol and violence overall. For example, policymakers should be cognisant that off-sales outlets–with increases in off-sale availability linked to levels of violence [52] and intimate partner violence [62]– have been demonstrated to be most densely clustered in the most deprived neighbourhoods [13]. Licensing applications in England and Wales currently must be considered on their individual merits in all but exceptional circumstances [63], meaning it is difficult for licensing authorities to address broader public health and crime prevention concerns such as this. Licensing practices in England and Wales should be revisited to address this, as has been advocated for by many in terms of public health considerations more generally [64, 65]. Similarly, despite the lowest SES groups drinking less on average, minimum unit pricing has been modelled to show promise in improving health outcomes for the lowest socioeconomic groups to the greatest degree [66] and to have potential to be implemented without raising concerns of regressivity [67]. As research has repeatedly linked the price of alcohol and levels of violence [68, 69], it should be investigated whether minimum unit pricing can further disproportionately benefit lower socioeconomic groups by reducing their alcohol-related violence victimisation levels.

This study has illuminated socioeconomic disparities in victimisation through alcohol-related domestic and acquaintance violence. These form part of a broader disparity in alcohol-related violence victimisation overall. While some suggestions for future research directions have been put forward, this finding is itself a notable contribution to our understanding of the unequal burden that alcohol harms place on the lowest SES groups. Along with action to address environmental and economic drivers of socioeconomic inequality, policymakers should address the provision of publicly funded domestic violence services in lower SES areas as a matter of urgency, coupled with action on the price and availability of alcohol, which have shown promise in beginning to ameliorate this imbalance.

## Supporting information

S1 File (DOCX)

## References

[1] Marmot M, Bell R. Fair society, healthy lives London: The Marmot Review, 2010.

[2] Bushman BJ, Cooper HM. Effects of alcohol on human aggression: An intergrative research review. Psychological bulletin. 1990;107(3):341 10.1037/0033-2909.107.3.341 2140902

[3] Boles SM, Miotto K. Substance abuse and violence: A review of the literature. Aggression and violent behavior. 2003;8(2):155–74.

[4] Lipsey MW, Wilson DB, Cohen MA, Derzon JH. Is there a causal relationship between alcohol use and violence? In: Galanter M, editor. Recent developments in alcoholism. New York: Kluwer Academic Publishers; 2002 p. 245–82. 10.1007/0-306-47141-8_14 9122498

[5] Boden JM, Fergusson DM, Horwood LJ. Alcohol misuse and violent behavior: Findings from a 30-year longitudinal study. Drug and alcohol dependence. 2012;122(1–2):135–41. 10.1016/j.drugalcdep.2011.09.023 22015176

[6] Office for National Statistics. The nature of violent crime in England and Wales: year ending March 2018; 2019 [cited 2020 30 May]. Available from: https://www.ons.gov.uk/peoplepopulationandcommunity/crimeandjustice/articles/thenatureofviolentcrimeinenglandandwales/yearendingmarch2018.

[7] Beard E, Brown J, West R, Angus C, Brennan A, Holmes J, et al Deconstructing the alcohol harm paradox: a population based survey of adults in England. PloS one. 2016;11(9). 10.1371/journal.pone.0160666 27682619 PMC5040414

[8] Lewer D, Meier P, Beard E, Boniface S, Kaner E. Unravelling the alcohol harm paradox: a population-based study of social gradients across very heavy drinking thresholds. BMC public health. 2016;16(1):599 10.1186/s12889-016-3265-9 27430342 PMC4950253

[9] Katikireddi SV, Whitley E, Lewsey J, Gray L, Leyland AH. Socioeconomic status as an effect modifier of alcohol consumption and harm: analysis of linked cohort data. The Lancet Public Health. 2017;2(6):e267–e76. 10.1016/S2468-2667(17)30078-6 28626829 PMC5463030

[10] Zinberg NE. Drug, set, and setting: The basis for controlled intoxicant use New Haven: Yale University Press; 1984.

[11] Graham K, Leonard KE, Room R, Wild TC, Pihl RO, Bois C, et al Current directions in research on understanding and preventing intoxicated aggression. Addiction. 1998;93(5):659–76. 10.1046/j.1360-0443.1998.9356593.x 9692266

[12] Graham K, Wells S. ‘Somebody’s gonna get their head kicked in tonight!’ Aggression among young males in bars—a question of values? British Journal of Criminology. 2003;43(3):546–66.

[13] Shortt NK, Tisch C, Pearce J, Mitchell R, Richardson EA, Hill S, et al A cross-sectional analysis of the relationship between tobacco and alcohol outlet density and neighbourhood deprivation. BMC public health. 2015;15(1):1014 10.1186/s12889-015-2321-1 26437967 PMC4595054

[14] Green S. Crime, victimisation and vulnerability In: Walklate S, editor. Handbook of victims and victimology. Cullompton: Willan; 2007 p. 107–34.

[15] Dignan J. Understanding victims and restorative justice Maidenhead: Open University Press; 2005.

[16] Brennan IR, Moore SC, Shepherd JP. Risk factors for violent victimisation and injury from six years of the British Crime Survey. International Review of Victimology. 2010;17(2):209–29.

[17] Budd T, Tedstone C, Curry D. Alcohol-related assault: findings from the British Crime Survey. London: Home Office, 2003.

[18] Conway DI, McMahon AD, Graham L, Snedker S, McCluskey K, Devlin M, et al The scar on the face of Scotland: deprivation and alcohol-related facial injuries in Scotland. Journal of Trauma and Acute Care Surgery. 2010;68(3):644–9. 10.1097/TA.0b013e3181a5ed18 19918199

[19] Quigg Z, Bellis MA, Grey H, Webster J, Hughes K. Alcohol’s harms to others in Wales, United Kingdom: Nature, magnitude and associations with mental well-being. Addictive behaviors reports. 2019;9:100162 10.1016/j.abrep.2019.100162 31193765 PMC6542752

[20] Breen C, Shakeshaft A, Slade T, Love S, D’Este C, Mattick RP. Do community characteristics predict alcohol-related crime? Alcohol and Alcoholism. 2011;46(4):464–70. 10.1093/alcalc/agr040 21546376

[21] Law Reiner R. and order: an honest citizen’s guide to crime and control. Malden: Polity Press; 2007.

[22] Graham K, Livingston M. The relationship between alcohol and violence–population, contextual and individual research approaches. Drug and Alcohol review. 2011;30(5):453 10.1111/j.1465-3362.2011.00340.x 21896066 PMC3170096

[23] Leonard KE. Alcohol and intimate partner violence: when can we say that heavy drinking is a contributing cause of violence? Addiction. 2005;100(4):422–5. 10.1111/j.1360-0443.2005.00994.x 15784050

[24] Jewkes R. Intimate partner violence: causes and prevention. The Lancet. 2002;359(9315):1423–9. 10.1016/S0140-6736(02)08357-5 11978358

[25] Greenfeld LA. Alcohol and crime: An analysis of national data on the prevalence of alcohol involvement in crime Washington, D.C.: US Department of Justice, 1998.

[26] Cogan R, Ballinger BC III. Alcohol problems and the differentiation of partner, stranger, and general violence. Journal of Interpersonal Violence. 2006;21(7):924–35. 10.1177/0886260506289177 16731992

[27] Walby S, Towers J, Francis B. Is violent crime increasing or decreasing? A new methodology to measure repeat attacks making visible the significance of gender and domestic relations. British Journal of Criminology. 2016;56(6):1203–34.

[28] Collins R. Violence: A micro-sociological theory Princeton: Princeton University Press; 2008.

[29] World Health Organisation. Interpersonal violence and alcohol. Geneva: World Health Organisation, 2006.

[30] Østergaard J, Andrade SB. Who pre-drinks before a night out and why? Socioeconomic status and motives behind young people’s pre-drinking in the United Kingdom. Journal of Substance Use. 2014;19(3):229–38.

[31] Lightowlers C. Heterogeneity in drinking practices in England and Wales and its association with violent behavior: A latent class analysis. Substance use & misuse. 2017;52(13):1721–32. 10.1080/10826084.2017.1307408 28704109

[32] Dodd T, Nicholas S, Povey D, Walker A. Crime in England and Wales 2003/2004. London: Home Office, 2004.

[33] Khalifeh H, Howard LM, Osborn D, Moran P, Johnson S. Violence against people with disability in England and Wales: findings from a national cross-sectional survey. PloS one. 2013;8(2). 10.1371/journal.pone.0055952 23437079 PMC3577814

[34] Mawby R, Walklate S. Critical victimology: International perspectives London: Sage Publications Ltd; 1994.

[35] Department for Environment Food and Rural Affairs. Rural poverty—to 2017/18. London: Department for Environment, Food and Rural Affairs, 2019.

[36] Office for National Statistics. Crime Survey for England and Wales 2013–2014. 3rd Edition ed. London: UK Data Service; 2020.

[37] Office for National Statistics. Crime Survey for England and Wales 2014–2015. 2nd Edition ed. London: UK Data Service; 2020.

[38] Office for National Statistics. Crime Survey for England and Wales 2015–2016. 2nd Edition ed. London: UK Data Service; 2020.

[39] Office for National Statistics. Crime Survey for England and Wales 2016–2017. 2nd Edition ed. London: UK Data Service; 2020.

[40] Office for National Statistics. Crime Survey for England and Wales 2017–2018. 2nd Edition ed. London: UK Data Service; 2020.

[41] Office for National Statistics. User guide to crime statistics for England and Wales. London: Office for National Statistics, 2020.

[42] Office For National Statistics. Crime in England and Wales QMI. London: Office For National Statistics, 2020.

[43] Walby S, Allen J. Domestic violence, sexual assault and stalking: Findings from the British Crime Survey. London: Home Office, 2004.

[44] Towers J. Economic Inequality and Intimate Partner Violence Against Women: An Analysis of the British Crime Survey 2008/09. Lancaster: Lancaster University; 2013.

[45] Boniface S, Lewer D, Hatch SL, Goodwin L. Associations between interrelated dimensions of socio-economic status, higher risk drinking and mental health in South East London: A cross-sectional study. PloS one. 2020;15(2):e0229093 10.1371/journal.pone.0229093 32059050 PMC7021306

[46] Tilley N, Tseloni A. Choosing and Using Statistical Sources in Criminology: What Can the Crime Survey for England and Wales Tell Us? Legal Information Management. 2016;16(2):78–90.

[47] UK Statistics Authority. Statistics on Crime in England and Wales. London: UK Statistics Authority, 2014.

[48] Davies P, Francis P, Greer C. Victims, Crime and Society: An Introduction. London: Sage Publications Ltd; 2017.

[49] Fone DL, Farewell DM, White J, Lyons RA, Dunstan FD. Socioeconomic patterning of excess alcohol consumption and binge drinking: a cross-sectional study of multilevel associations with neighbourhood deprivation. BMJ open. 2013;3(4):e002337 10.1136/bmjopen-2012-002337 23587771 PMC3641461

[50] Mulia N, Karriker-Jaffe KJ. Interactive influences of neighborhood and individual socioeconomic status on alcohol consumption and problems. Alcohol and alcoholism. 2012;47(2):178–86. 10.1093/alcalc/agr168 22262507 PMC3284688

[51] Grittner U, Kuntsche S, Gmel G, Bloomfield K. Alcohol consumption and social inequality at the individual and country levels—results from an international study. The European Journal of Public Health. 2013;23(2):332–9. 10.1093/eurpub/cks044 22562712 PMC3610336

[52] Alcohol Focus Scotland and Centre for Research on Environment Society and Health at the Universities of Edinburgh and Glasgow. Alcohol Outlet Availability and Harm in Scotland. Glasgow: Alcohol Focus Scotland; 2018.

[53] Hague G, Thiara R, Mullender A. Disabled women, domestic violence and social care: the risk of isolation, vulnerability and neglect. British Journal of Social Work. 2011;41(1):148–65.

[54] Balderston S. Surviving Disablist Hate Rape: Barriers, Intersectionalities and Collective Interventions with Disabled Women in the North of England. Lancaster: Lancaster University; 2014.

[55] Viermo V. Violence Against Disabled Women In: Kristiansen K, Traustadottir R, editors. Gender and Disability Research in the Nordic Countries. Lund: Student Litteratur; 2004 p. 327–46.

[56] Finney A. Violence in the night-time economy: Key findings from the research London: Home Office; 2004.

[57] National Pub Watch. Welcome to National Pubwatch. n.d. Available from: https://www.nationalpubwatch.org.uk/.

[58] Best Bar None. What is Best Bar None? 2020. Available from: https://bbnuk.com/.

[59] Alcohol Concern, Alcohol Research UK. The Hardest Hit: Addressing the Crisis in Alcohol Treatment Services. London: Alcohol Concern and Alcohol Research UK; 2018.

[60] Holly J. Mapping the Maze: Services for women experiencing multiple disadvantage in England and Wales. London: Agenda & AVA; 2017.

[61] The National Institute for Health and Care Excellence. Domestic violence and abuse: multi-agency working. London: The National Institute for Health and Care Excellence; 2014.

[62] Livingston M. A longitudinal analysis of alcohol outlet density and domestic violence. Addiction. 2011;106(5):919–25. 10.1111/j.1360-0443.2010.03333.x 21205052

[63] Office Home. Revised Guidance Issued Under Section 182 of the Licensing Act 2003. London: HM Government; 2018.

[64] Anderson W, Gilmore SI, Bauld L, Bellis M, Brown KA, Drummond C, et al Health First: An evidence-based alcohol strategy for the UK. Stirling: University of Stirling; 2013.

[65] Morleo M, Lightowlers C, Anderson Z, Cook PA, Harkins C, Bellis MA. A review of the impact of the Licensing Act 2003 on levels of violence in England and Wales: a public health perspective. Crime Prevention and Community Safety. 2009;11(3):204–18.

[66] Angus C, Holmes J, Pryce R, Meier P, Brennan A. Alcohol and cancer trends: Intervention Studies. London: University of Sheffield and Cancer Research UK; 2016.

[67] Connolly K, Lisenkova K, McGregor P. The Economic Impact of Changes in Alcohol Consumption in the UK. Glasgow: Fraser of Allander Institute and the Institute of Alcohol Studies, 2018.

[68] Booth A, Meier P, Shapland J, Wong R, Paisley S. Alcohol pricing and criminal harm: a rapid evidence assessment of the published research literature Sheffield: University of Sheffield, 2011.

[69] Office Home. The Likely Impacts of Increasing Alcohol Price: A Summary Review of the Evidence Base. London: Home Office, 2011.

