# Supplementary material for: The socioeconomic distribution of alcohol-related violence in England and Wales
Source: PLoS One. 2021 Feb 18;16(2):e0243206. doi: 10.1371/journal.pone.0243206 (PMC7891736; doi:10.1371/journal.pone.0243206)
Supplement: S1 File — (DOCX) [file pone.0243206.s001.docx]

**S1 File**

**S1 Table: Respondent sex**

|  | Frequency | Percent |
| --- | --- | --- |
| Male | 112166251 | 48.9 |
| Female | 117213372 | 51.1 |
| Total | 229379622 | 100.0 |

*Base (n=174178 unweighted) = whole sample.*

**S2 Table: Respondent age**

|  | Frequency | Percent |
| --- | --- | --- |
| 30 and under | 54227428 | 23.7 |
| Over 30 | 174372664 | 76.3 |
| Total | 228600091 | 100.0 |

*Base (n=* *173511 unweighted) = whole sample, excluding those marked missing (n=667, unweighted, 0.4% of whole sample), converted to a binary variable to maintain statistical power (those over 30 years and those 30 years and below).*

**S3 Table: Whether the respondent lives in a rural or urban area**

|  | Frequency | Percent |
| --- | --- | --- |
| Rural | 42655560 | 18.6 |
| Urban | 186724062 | 81.4 |
| Total | 229379622 | 100.0 |

*Base (n=174178 unweighted) = whole sample.*

**S4 Table: Whether respondent has a disability**

|  | Frequency | Percent |
| --- | --- | --- |
| No long standing illness | 180041437 | 78.8 |
| Long standing illness | 48498142 | 21.2 |
| Total | 228539579 | 100.0 |

*Base (n=173481, unweighted) = whole sample, excluding those marked missing (n=697, unweighted, 0.4% of whole sample).*

This is a derived variable published in CSEW data, Disability/long-standing illness ONS, which is derived from the following survey items:

"Do you have any physical or mental health conditions or illnesses lasting or expected to last for 12 months or more? 1. Yes; 2. No”

Do any of these conditions or illnesses affect you in any of the areas shown on this card? 1. Vision (for example, blindness or partial sight); 2. Hearing (for example, deafness or partial hearing); 3. Mobility (for example, walking short distances or climbing stairs); 4. Dexterity (for example, lifting and carrying objects, using a keyboard); 5. Learning or understanding or concentrating; 6. Memory; 7. Mental health; 8. Stamina or breathing or fatigue; 9. Socially or behaviourally (for example associated with autism, attention deficit disorder or Asperger's syndrome); 10. Other (SPECIFY); 11. SPONTANEOUS ONLY: None of the above” [1 p. 210]

**S5 Table: Visited club in the last month**

|  | Frequency | Percent |
| --- | --- | --- |
| No club in last month | 207883065 | 91.0 |
| Club in last month | 20646323 | 9.0 |
| Total | 228529388 | 100.00 |

*Base (n=173800, unweighted) = whole sample, excluding those marked missing (n=378, unweighted, 0.2% of whole sample).*

**S6 Table: Frequency of visiting pubs in last month**

|  | Frequency | Percent |
| --- | --- | --- |
| Less than once a week | 184066894 | 80.3 |
| Weekly or more | 45192650 | 19.7 |
| Total | 229259544 | 100.0 |

*Base (n=174090, unweighted) = whole sample, excluding those marked missing (n=88, unweighted, 0.1% of whole sample), converted to a binary variable to maintain statistical power (those visiting pub weekly or more, and those visiting the pub less than weekly (including no visits)).*

**S7 Table. Binomial logistic regression of association between socioeconomic status (measured by total household income), demographic and violence risk factor variables, and experience of alcohol-related violence and subtypes, run on unweighted data**

| Alcohol-related violence | | | | |
| --- | --- | --- | --- | --- |
|  | Experienced alcohol-related violence? | Experienced alcohol-related domestic violence? | Experienced alcohol-related stranger violence? | Experienced alcohol-related acquaintance violence? |
| Female | β = -0.568  SE = 0.061  OR = 0.566 (95% CI 0.503-0.638)  p<0.001* | β = 0.744  SE = 0.155  OR = 2.104 (95% CI 1.553-2.851)  p<0.001* | β = -1.223  SE = 0.098  OR = 0.294 (95% CI 0.243-0.357)  p<0.001* | β = -0.416  SE = 0.101  OR = 0.660 (95% CI 0.541-0.804)  p<0.001* |
| Urban | β = 0.086  SE = 0.077  OR = 1.090 (95% CI 0.937-1.269)  p=0.265 | β = -0.205  SE = 0.166  OR = 0.815 (95% CI 0.589-1.127)  p=0.216 | β = 0.213  SE = 0.115  OR =1.237 (95% CI 0.987-1.551)  p=0.064 | β = 0.04  SE = 0.129  OR = 1.041 (95% CI 0.808-1.341)  p=0.755 |
| Age (over 30) | β = -1.242  SE = 0.069  OR = 0.289 (95% CI 0.252-0.330)  p<0.001* | β = -1.001  SE = 0.166  OR = 0.367 (95% CI 0.265-0.508)  p<0.001* | β = -1.285  SE = 0.098  OR = 0.277 (95% CI 0.228-0.335)  p<0.001* | β = -1.277  SE = 0.116  OR = 0.279 (95% CI 0.222-0.35)  p<0.001* |
| Visits pub weekly or more | β = 0.361  SE = 0.068  OR = 1.435 (95% CI 1.255-1.641)  p<0.001* | β = 0.266  SE = 0.180  OR = 1.304 (95% CI 0.916-1.857)  p=0.141 | β = 0.427  SE = 0.093  OR = 1.532 (95% CI 1.278-1.837)  p<0.001* | β = 0.281  SE = 0.12  OR = 1.325 (95% CI 1.047-1.676)  p=0.019 |
| Visited nightclub/disco in last month | β = 1.040  SE = 0.079  OR = 2.829 (95% CI 2.424-3.302)  p<0.001* | β = 0.726  SE = 0.212  OR = 2.067 (95% CI 1.364-3.133)  p=0.001* | β = 1.258  SE = 0.105  OR = 3.518 (95% CI 2.862-4.325)  p<0.001* | β = 0.832  SE = 0.14  OR = 2.298 (95% CI 1.748-3.022)  p<0.001* |
| Disability or long-standing illness | β = 0.675  SE = 0.068  OR = 1.963 (95% CI 1.719-2.241)  p<0.001* | β = 1.037  SE = 0.146  OR = 2.822 (95% CI 2.119-3.758)  p<0.001* | β = 0.267  SE = 0.109  OR = 1.307 (95% CI 1.055-1.619)  p=0.014 | β = 0.917  SE = 0.108  OR = 2.503 (95% CI 2.023-3.095)  p<0.001* |
| Total household income: £19,999 and under | β = 0.251  SE = 0.075  OR = 1.286 (95% CI 1.110-1.489)  p=0.001* | β = 0.919  SE = 0.206  OR = 2.508 (95% CI 1.675-3.754)  p<0.001* | β = -0.199  SE = 0.104  OR = 0.819 (95% CI 0.668-1.005)  p=0.056 | β = 0.646  SE = 0.139  OR = 1.908 (95% CI 1.454-2.504)  p<0.001* |
| Total household income: £20,000 to £39,999 | β = 0.063  SE = 0.08  OR = 1.066 (95% CI 0.911-1.246)  p=0.426 | β = 0.320  SE = 0.232  OR = 1.377 (95% CI 0.874-2.168)  p=0.168 | β = -0.118  SE = 0.103  OR = 0.889 (95% CI 0.726-1.089)  p=0.255 | β = 0.4  SE = 0.148  OR = 1.492 (95% CI 1.117-1.992)  p=0.007 |
| Total household income: £40,000 and above | Ref | Ref | Ref | Ref |

*Experienced alcohol-related violence: Yes, n = 1179, unweighted; Experienced alcohol-related domestic violence: Yes, n = 213, unweighted; Experienced alcohol-related stranger violence: Yes, n = 580, unweighted; Experienced alcohol-related acquaintance violence: Yes, n = 410, unweighted. Base for all, n = 150570, unweighted = whole sample excluding respondents marked missing for total household income variable or other demographic and violence risk factor variables (n=23608, 13.6% of whole sample). *Significant based on threshold adjusted through Bonferroni correction to p<0.004, from an original value of p<0.05.*

**S8 Table. Binomial logistic regression of association between socioeconomic status (measured by housing tenure), demographic and violence risk factor variables, and experience of alcohol-related violence and subtypes, run on unweighted data**

| Alcohol-related violence | | | | |
| --- | --- | --- | --- | --- |
|  | Experienced alcohol-related violence? | Experienced alcohol-related domestic violence? | Experienced alcohol-related stranger violence? | Experienced alcohol-related acquaintance violence? |
| Female | β = -0.586  SE = 0.058  OR = 0.557 (95% CI 0.497-0.624)  p<0.001* | β = 0.745  SE = 0.149  OR = 2.106 (95% CI 1.573-2.819)  p<0.001* | β = -1.238  SE = 0.093  OR = 0.290 (95% CI 0.242-0.348)  p<0.001* | β = -0.416  SE = 0.097  OR = 0.659 (95% CI 0.545-0.798)  p<0.001* |
| Urban | β = 0.010  SE = 0.074  OR = 1.010 (95% CI 0.873-1.167)  p=0.896 | β = -0.235  SE = 0.163  OR = 0.791 (95% CI 0.574-1.089)  p=0.150 | β = 0.134  SE = 0.108  OR = 1.143 (95% CI 0.924-1.414)  p=0.218 | β = -0.071  SE = 0.123  OR = 0.931 (95% CI 0.732-1.185)  p=0.562 |
| Age (over 30) | β = -1.085  SE = 0.069  OR = 0.338 (95% CI 0.295-0.387)  p<0.001* | β = -0.757  SE = 0.164  OR = 0.469 (95% CI 0.341-0.647)  p<0.001* | β = -1.177  SE = 0.098  OR = 0.308 (95% CI 0.254-0.374)  p<0.001* | β = -1.073  SE = 0.117  OR = 0.342 (95% CI 0.272-0.430)  p<0.001* |
| Visits pub weekly or more | β = 0.448  SE = 0.066  OR = 1.566 (95% CI 1.377-1.781)  p<0.001* | β = 0.340  SE = 0.174  OR = 1.405 (95% CI 0.999-1.975)  p=0.050 | β = 0.502  SE = 0.088  OR = 1.652 (95% CI 1.390-1.965)  p<0.001* | β = 0.373  SE = 0.115  OR = 1.452 (95% CI 1.158-1.820)  p=0.001* |
| Visited nightclub/disco in last month | β = 1.010  SE = 0.076  OR = 2.745 (95% CI 2.367-3.184)  p<0.001* | β = 0.662  SE = 0.202  OR = 1.938 (95% CI 1.304-2.882)  p=0.001* | β = 1.221  SE = 0.101  OR = 3.391 (95% CI 2.782-4.134)  p<0.001* | β = 0.825  SE = 0.134  OR = 2.282 (95% CI 1.755-2.967)  p<0.001* |
| Disability or long-standing illness | β = 0.574  SE = 0.065  OR = 1.776 (95% CI 1.564-2.016)  p<0.001* | β = 0.995  SE = 0.141  OR = 2.704 (95% CI 2.051-3.565)  p<0.001* | β = 0.128  SE = 0.105  OR = 1.137 (95% CI 0.926-1.396)  p=0.221 | β = 0.842  SE = 0.105  OR = 2.322 (95% CI 1.892-2.850)  p<0.001* |
| Housing tenure: social renters | β = 0.879  SE = 0.073  OR = 2.409 (95% CI 2.087-2.781)  p<0.001* | β = 1.586  SE = 0.172  OR = 4.883 (95% CI 3.489-6.835)  p<0.001* | β = 0.363  SE = 0.115  OR = 1.438 (95% CI 1.148-1.801)  p=0.002* | β = 1.140  SE = 0.121  OR = 3.128 (95% CI 2.468-3.963)  p<0.001* |
| Housing tenure: private renters | β = 0.574  SE = 0.072  OR = 1.775 (95% CI 1.541-2.045)  p<0.001* | β = 1.176  SE = 0.187  OR = 3.241 (95% CI 2.245-4.679)  p<0.001* | β = 0.322  SE = 0.097  OR = 1.379 (95% CI 1.141-1.668)  p=0.001* | β = 0.708  SE = 0.127  OR = 2.029 (95% CI 1.581-2.604)  p<0.001* |
| Housing tenure: owners | Ref | Ref | Ref | Ref |

*Experienced alcohol-related violence: Yes, n = 1286, unweighted; Experienced alcohol-related domestic violence: Yes, n = 230, unweighted; Experienced alcohol-related stranger violence: Yes, n = 639, unweighted; Experienced alcohol-related acquaintance violence: Yes, n = 443, unweighted. Base for all, n = 171775, unweighted = whole sample excluding respondents marked missing for housing tenure variable or other demographic and violence risk factor variables (n=2403, 1.4% of whole sample). *Significant based on threshold adjusted through Bonferroni correction to p<0.004, from an original value of p<0.05.*

**S9 Table. Binomial logistic regression of association between socioeconomic status (measured by occupation), demographic and violence risk factor variables, and experience of alcohol-related violence and subtypes, run on unweighted data**

| Alcohol-related violence | | | | |
| --- | --- | --- | --- | --- |
|  | Experienced alcohol-related violence? | Experienced alcohol-related domestic violence? | Experienced alcohol-related stranger violence? | Experienced alcohol-related acquaintance violence? |
| Female | β = -0.562  SE = 0.061  OR = 0.570 (95% CI 0.506-0.642)  p<0.001* | β = 0.709  SE = 0.149  OR = 2.032 (95% CI 1.516-2.723)  p<0.001* | β = -1.233  SE = 0.097  OR = 0.291 (95% CI 0.241-0.352)  p<0.001* | β = -0.382  SE = 0.101  OR = 0.683 (95% CI 0.560-0.833)  p<0.001* |
| Urban | β = 0.084  SE = 0.076  OR = 1.087 (95% CI 0.938-1.261)  p=0.267 | β = -0.102  SE = 0.164  OR = 0.903 (95% CI 0.654-1.245)  p=0.533 | β = 0.187  SE = 0.112  OR = 1.205 (95% CI 0.969-1.5)  p=0.094 | β = 0.013  SE = 0.126  OR = 1.013 (95% CI 0.792-1.297)  p=0.916 |
| Age (over 30) | β = -1.300  SE = 0.070  OR = 0.273 (95% CI 0.238-0.312)  p<0.001* | β = -1.127  SE = 0.163  OR = 0.324 (95% CI 0.235-0.446)  p<0.001* | β = -1.349  SE = 0.098  OR = 0.259 (95% CI 0.214-0.315)  p<0.001* | β = -1.275  SE = 0.119  OR = 0.280 (95% CI 0.221-0.353)  p<0.001* |
| Visits pub weekly or more | β = 0.407  SE = 0.068  OR = 1.503 (95% CI 1.315-1.717)  p<0.001* | β = 0.195  SE = 0.178  OR = 1.215 (95% CI 0.857-1.721)  p=0.274 | β = 0.477  SE = 0.092  OR = 1.611 (95% CI 1.345-1.930)  p<0.001* | β = 0.367  SE = 0.119  OR = 1.443 (95% CI 1.142-1.823)  p=0.002* |
| Visited nightclub/disco in last month | β = 0.996  SE = 0.08  OR = 2.707 (95% CI 2.312-3.169)  p<0.001* | β = 0.809  SE = 0.207  OR = 2.246 (95% CI 1.498-3.369)  p<0.001* | β = 1.141  SE = 0.108  OR = 3.130 (95% CI 2.535-3.864)  p<0.001* | β = 0.873  SE = 0.143  OR = 2.395 (95% CI 1.808-3.172)  p<0.001* |
| Disability or long-standing illness | β = 0.636  SE = 0.066  OR = 1.889 (95% CI 1.661-2.149)  p<0.001* | β = 1.093  SE = 0.14  OR = 2.983 (95% CI 2.266-3.926)  p<0.001* | β = 0.145  SE = 0.107  OR = 1.156 (95% CI 0.938-1.426)  p=0.175 | β = 0.933  SE = 0.106  OR = 2.542 (95% CI 2.064-3.131)  p<0.001* |
| Occupation: never worked or long term unemployed | β = 0.517  SE = 0.139  OR =1.677 (95% CI 1.278-2.201)  p<0.001* | β = 0.689  SE = 0.287  OR = 1.993 (95% CI 1.135-3.497)  p=0.016 | β = 0.163  SE = 0.226  OR = 1.177 (95% CI 0.756-1.833)  p=0.470 | β = 0.880  SE = 0.228  OR = 2.412 (95% CI 1.543-3.769)  p<0.001* |
| Occupation: routine or manual occupation | β = 0.323  SE = 0.072  OR = 1.382 (95% CI 1.199-1.592)  p<0.001* | β = 0.581  SE = 0.171  OR = 1.788 (95% CI 1.279-2.498)  p=0.001* | β = 0.024  SE = 0.1  OR = 1.024 (95% CI 0.842-1.245)  p=0.812 | β = 0.689  SE = 0.133  OR = 1.991 (95% CI 1.534-2.585)  p<0.001* |
| Occupation: intermediate occupation | β = 0.327  SE = 0.081  OR = 1.387 (95% CI 1.183-1.626)  p<0.001* | β = 0.145  SE = 0.206  OR = 1.156 (95% CI 0.772-1.729)  p=0.482 | β = 0.205  SE = 0.11  OR = 1.228 (95% CI 0.990-1.523)  p=0.062 | β = 0.683  SE = 0.146  OR = 1.980 (95% CI 1.486-2.638)  p<0.001* |
| Occupation: managerial or professional occupation | Ref | Ref | Ref | Ref |

*Experienced alcohol-related violence: Yes, n = 1194, unweighted; Experienced alcohol-related domestic violence: Yes, n = 224, unweighted; Experienced alcohol-related stranger violence: Yes, n = 586, unweighted; Experienced alcohol-related acquaintance violence: Yes, n = 408, unweighted. Base for all, n = 166034, unweighted = whole sample excluding respondents marked missing for occupation variable or other demographic and violence risk factor variables (n=8144, 4.7% of whole sample). *Significant based on threshold adjusted through Bonferroni correction to p<0.004, from an original value of p<0.05.*

**S10 Table. Binomial logistic regression of association between socioeconomic status (measured by total household income), demographic and violence risk factor variables, and experience of alcohol-related violence and subtypes, run on weighted data, full results**

| Alcohol-related violence | | | | |
| --- | --- | --- | --- | --- |
|  | Experienced alcohol-related violence? | Experienced alcohol-related domestic violence? | Experienced alcohol-related stranger violence? | Experienced alcohol-related acquaintance violence? |
| Female | β = -0.697  SE = 0.002  OR = 0.498 (95% CI 0.496-0.500)  p<0.001* | β = 0.568  SE = 0.004  OR = 1.765 (95% CI 1.750-1.781)  p<0.001* | β = -1.247  SE = 0.003  OR = 0.287 (95% CI 0.286-0.289)  p<0.001* | β = -0.453  SE = 0.003  OR = 0.636 (95% CI 0.633-0.639)  p<0.001* |
| Urban | β = 0.038  SE = 0.002  OR = 1.039 (95% CI 1.034-1.043)  p<0.001* | β = -0.043  SE = 0.006  OR = 0.958 (95% CI 0.948-0.969)  p<0.001* | β = 0.147  SE = 0.003  OR = 1.158 (95% CI 1.151-1.165)  p<0.001* | β = -0.149  SE = 0.004  OR = 0.861 (95% CI 0.855-0.867)  p<0.001* |
| Age (over 30) | β = -1.154  SE = 0.002  OR = 0.315 (95% CI 0.314-0.316)  p<0.001* | β = -0.744  SE = 0.005  OR = 0.475 (95% CI 0.471-0.480)  p<0.001* | β = -1.205  SE = 0.002  OR = 0.300 (95% CI 0.298-0.301)  p<0.001* | β = -1.246  SE = 0.003  OR = 0.288 (95% CI 0.286-0.289)  p<0.001* |
| Visits pub weekly or more | β = 0.407  SE = 0.002  OR = 1.502 (95% CI 1.497-1.507)  p<0.001* | β = 0.351  SE = 0.005  OR = 1.421 (95% CI 1.406-1.436)  p<0.001* | β = 0.475  SE = 0.002  OR = 1.609 (95% CI 1.601-1.616)  p<0.001* | β = 0.314  SE = 0.003  OR = 1.369 (95% CI 1.361-1.377)  p<0.001* |
| Visited nightclub/disco in last month | β = 1.026  SE = 0.002  OR = 2.790 (95% CI 2.780-2.801)  p<0.001* | β = 0.572  SE = 0.006  OR = 1.772 (95% CI 1.751-1.794)  p<0.001* | β = 1.188  SE = 0.003  OR = 3.281 (95% CI 3.265-3.297)  p<0.001* | β = 0.889  SE = 0.003  OR = 2.433 (95% CI 2.417-2.449)  p<0.001* |
| Disability or long-standing illness | β = 0.660  SE = 0.002  OR = 1.935 (95% CI 1.928-1.942)  p<0.001* | β = 1.143  SE = 0.004  OR = 3.138 (95% CI 3.110-3.165)  p<0.001* | β = 0.205  SE = 0.003  OR = 1.228 (95% CI 1.221-1.235)  p<0.001* | β = 1.008  SE = 0.003  OR = 2.740 (95% CI 2.725-2.756)  p<0.001* |
| Total household income: £19,999 and under | β = 0.239  SE = 0.002  OR = 1.270 (95% CI 1.265-1.274)  p<0.001* | β = 0.877  SE = 0.006  OR = 2.403 (95% CI 2.376-2.430)  p<0.001* | β = -0.093  SE = 0.003  OR = 0.911 (95% CI 0.907-0.916)  p<0.001* | β = 0.547  SE = 0.003  OR = 1.729 (95% CI 1.717-1.740)  p<0.001* |
| Total household income: £20,000 to £39,999 | β = 0.125  SE = 0.002  OR = 1.133 (95% CI 1.128-1.137)  p<0.001* | β = 0.302  SE = 0.006  OR = 1.352 (95% CI 1.335-1.369)  p<0.001* | β = -0.019  SE = 0.003  OR = 0.981 (95% CI 0.976-0.986)  p<0.001* | β = 0.412  SE = 0.004  OR = 1.510 (95% CI 1.499-1.520)  p<0.001* |
| Total household income: £40,000 and above | Ref | Ref | Ref | Ref |

*Experienced alcohol-related violence: Yes, n = 1179, unweighted; Experienced alcohol-related domestic violence: Yes, n = 213, unweighted; Experienced alcohol-related stranger violence: Yes, n = 580, unweighted; Experienced alcohol-related acquaintance violence: Yes, n = 410, unweighted. Base for all, n = 150570, unweighted = whole sample excluding respondents marked missing for total household income variable or other demographic and violence risk factor variables (n=23608, 13.6% of whole sample). *Significant based on threshold adjusted through Bonferroni correction to p<0.004, from an original value of p<0.05.*

**S11 Table. Binomial logistic regression of association between socioeconomic status (measured by housing tenure), demographic and violence risk factor variables, and experience of alcohol-related violence and subtypes, run on weighted data, full results**

| Alcohol-related violence | | | | |
| --- | --- | --- | --- | --- |
|  | Experienced alcohol-related violence? | Experienced alcohol-related domestic violence? | Experienced alcohol-related stranger violence? | Experienced alcohol-related acquaintance violence? |
| Female | β = -0.716  SE = 0.002  OR = 0.489 (95% CI 0.487-0.490)  p<0.001* | β = 0.661  SE = 0.004  OR = 1.937 (95% CI 1.921-1.953)  p<0.001* | β = -1.269  SE = 0.002  OR = 0.281 (95% CI 0.280-0.283)  p<0.001* | β = -0.477  SE = 0.003  OR = 0.620 (95% CI 0.617-0.624)  p<0.001* |
| Urban | β = -0.020  SE = 0.002  OR = 0.980 (95% CI 0.976-0.984)  p<0.001* | β = -0.045  SE = 0.006  OR = 0.956 (95% CI 0.946-0.966)  p<0.001* | β = 0.092  SE = 0.003  OR = 1.097 (95% CI 1.091-1.103)  p<0.001* | β = -0.253  SE = 0.003  OR = 0.776 (95% CI 0.771-0.781)  p<0.001* |
| Age (over 30) | β = -1.074  SE = 0.002  OR = 0.342 (95% CI 0.340-0.343)  p<0.001* | β = -0.717  SE = 0.005  OR = 0.488 (95% CI 0.483-0.493)  p<0.001* | β = -1.148  SE = 0.002  OR = 0.317 (95% CI 0.316-0.319)  p<0.001* | β = -1.083  SE = 0.003  OR = 0.339 (95% CI 0.337-0.341)  p<0.001* |
| Visits pub weekly or more | β = 0.451  SE = 0.002  OR = 1.571 (95% CI 1.565-1.576)  p<0.001* | β = 0.455  SE = 0.005  OR = 1.576 (95% CI 1.561-1.592)  p<0.001* | β = 0.497  SE = 0.002  OR = 1.643 (95% CI 1.636-1.650)  p<0.001* | β = 0.358  SE = 0.003  OR = 1.430 (95% CI 1.422-1.438)  p<0.001* |
| Visited nightclub/disco in last month | β = 1.024  SE = 0.002  OR = 2.783 (95% CI 2.773-2.794)  p<0.001* | β = 0.593  SE = 0.006  OR = 1.809 (95% CI 1.789-1.830)  p<0.001* | β = 1.167  SE = 0.002  OR = 3.211 (95% CI 3.196-3.227)  p<0.001* | β = 0.903  SE = 0.003  OR = 2.468 (95% CI 2.452-2.484)  p<0.001* |
| Disability or long-standing illness | β = 0.580  SE = 0.002  OR = 1.786 (95% CI 1.780-1.793)  p<0.001* | β = 1.189  SE = 0.004  OR = 3.284 (95% CI 3.257-3.312)  p<0.001* | β = 0.103  SE = 0.003  OR = 1.108 (95% CI 1.102-1.114)  p<0.001* | β = 0.928  SE = 0.003  OR = 2.529 (95% CI 2.515-2.543)  p<0.001* |
| Housing tenure: social renters | β = 0.750  SE = 0.002  OR = 2.117 (95% CI 2.109-2.126)  p<0.001* | β = 1.302  SE = 0.005  OR = 3.678 (95% CI 3.641-3.715)  p<0.001* | β = 0.371  SE = 0.003  OR = 1.449 (95% CI 1.440-1.457)  p<0.001* | β = 1.035  SE = 0.003  OR = 2.816 (95% CI 2.797-2.834)  p<0.001* |
| Housing tenure: private renters | β = 0.370  SE = 0.002  OR = 1.448 (95% CI 1.443-1.453)  p<0.001* | β = 0.831  SE = 0.005  OR = 2.295 (95% CI 2.271-2.320)  p<0.001* | β = 0.199  SE = 0.002  OR = 1.220 (95% CI 1.215-1.226)  p<0.001* | β = 0.526  SE = 0.003  OR = 1.692 (95% CI 1.681-1.702)  p<0.001* |
| Housing tenure: owners | Ref | Ref | Ref | Ref |

*Experienced alcohol-related violence: Yes, n = 1286, unweighted; Experienced alcohol-related domestic violence: Yes, n = 230, unweighted; Experienced alcohol-related stranger violence: Yes, n = 639, unweighted; Experienced alcohol-related acquaintance violence: Yes, n = 443, unweighted. Base for all, n = 171775, unweighted = whole sample excluding respondents marked missing for housing tenure variable or other demographic and violence risk factor variables (n=2403, 1.4% of whole sample). *Significant based on threshold adjusted through Bonferroni correction to p<0.004, from an original value of p<0.05.*

**S12 Table. Binomial logistic regression of association between socioeconomic status (measured by occupation), demographic and violence risk factor variables, and experience of alcohol-related violence and subtypes, run on weighted data, full results**

| Alcohol-related violence | | | | |
| --- | --- | --- | --- | --- |
|  | Experienced alcohol-related violence? | Experienced alcohol-related domestic violence? | Experienced alcohol-related stranger violence? | Experienced alcohol-related acquaintance violence? |
| Female | β = -0.665  SE = 0.002  OR = 0.514 (95% CI 0.513-0.516)  p<0.001* | β = 0.562  SE = 0.004  OR = 1.755 (95% CI 1.740-1.769)  p<0.001* | β = -1.249  SE = 0.003  OR = 0.287 (95% CI 0.285-0.288)  p<0.001* | β = -0.371  SE = 0.003  OR = 0.690 (95% CI 0.686-0.694)  p<0.001* |
| Urban | β = 0.062  SE = 0.002  OR = 1.064 (95% CI 1.060-1.069)  p<0.001* | β = 0.116  SE = 0.006  OR = 1.123 (95% CI 1.111-1.136)  p<0.001* | β = 0.161  SE = 0.003  OR = 1.175 (95% CI 1.168-1.182)  p<0.001* | β = -0.173  SE = 0.003  OR = 0.841 (95% CI 0.836-0.847)  p<0.001* |
| Age (over 30) | β = -1.255  SE = 0.002  OR = 0.285 (95% CI 0.284-0.286)  p<0.001* | β = -0.999  SE = 0.005  OR = 0.368 (95% CI 0.365-0.372)  p<0.001* | β = -1.321  SE = 0.002  OR = 0.267 (95% CI 0.266-0.268)  p<0.001* | β = -1.239  SE = 0.003  OR = 0.290 (95% CI 0.288-0.291)  p<0.001* |
| Visits pub weekly or more | β = 0.449  SE = 0.002  OR = 1.566 (95% CI 1.561-1.572)  p<0.001* | β = 0.308  SE = 0.005  OR = 1.360 (95% CI 1.347-1.374)  p<0.001* | β = 0.481  SE = 0.002  OR = 1.618 (95% CI 1.611-1.625)  p<0.001* | β = 0.446  SE = 0.003  OR = 1.562 (95% CI 1.552-1.572)  p<0.001* |
| Visited nightclub/disco in last month | β = 0.934  SE = 0.002  OR = 2.544 (95% CI 2.534-2.554)  p<0.001* | β = 0.743  SE = 0.006  OR = 2.102 (95% CI 2.078-2.127)  p<0.001* | β = 1.045  SE = 0.003  OR = 2.844 (95% CI 2.830-2.859)  p<0.001* | β = 0.821  SE = 0.004  OR = 2.273 (95% CI 2.256-2.289)  p<0.001* |
| Disability or long-standing illness | β = 0.610  SE = 0.002  OR = 1.841 (95% CI 1.834-1.847)  p<0.001* | β = 1.190  SE = 0.004  OR = 3.286 (95% CI 3.258-3.314)  p<0.001* | β = 0.129  SE = 0.003  OR = 1.137 (95% CI 1.131-1.144)  p<0.001* | β = 0.968  SE = 0.003  OR = 2.634 (95% CI 2.618-2.649)  p<0.001* |
| Occupation: never worked or long term unemployed | β = 0.376  SE = 0.004  OR = 1.456 (95% CI 1.445-1.467)  p<0.001* | β = 0.496  SE = 0.009  OR = 1.642 (95% CI 1.613-1.671)  p<0.001* | β = 0.067  SE = 0.006  OR = 1.069 (95% CI 1.057-1.081)  p<0.001* | β = 0.779  SE = 0.006  OR = 2.180 (95% CI 2.153-2.207)  p<0.001* |
| Occupation: routine or manual occupation | β = 0.262  SE = 0.002  OR = 1.299 (95% CI 1.294-1.304)  p<0.001* | β = 0.517  SE = 0.005  OR = 1.677 (95% CI 1.660-1.693)  p<0.001* | β = 0.013  SE = 0.003  OR = 1.013 (95% CI 1.008-1.018)  p<0.001* | β = 0.642  SE = 0.004  OR = 1.900 (95% CI 1.887-1.914)  p<0.001* |
| Occupation: intermediate occupation | β = 0.339  SE = 0.002  OR = 1.403 (95% CI 1.397-1.409)  p<0.001* | β = 0.061  SE = 0.006  OR = 1.063 (95% CI 1.050-1.076)  p<0.001* | β = 0.283  SE = 0.003  OR = 1.326 (95% CI 1.319-1.334)  p<0.001* | β = 0.605  SE = 0.004  OR = 1.832 (95% CI 1.817-1.846)  p<0.001* |
| Occupation: managerial or professional occupation | Ref | Ref | Ref | Ref |

*Experienced alcohol-related violence: Yes, n = 1194, unweighted; Experienced alcohol-related domestic violence: Yes, n = 224, unweighted; Experienced alcohol-related stranger violence: Yes, n = 586, unweighted; Experienced alcohol-related acquaintance violence: Yes, n = 408, unweighted. Base for all, n = 166034, unweighted = whole sample excluding respondents marked missing for occupation variable or other demographic and violence risk factor variables (n=8144, 4.7% of whole sample). *Significant based on threshold adjusted through Bonferroni correction to p<0.004, from an original value of p<0.05.*

**REFERENCES**

1. Office For National Statistics, Kantar Public. 2017-18 Crime Survey for England and Wales Questionnaire (from April 2017). London: Office For National Statistics and Kantar Public; 2017.
